# All-Cause and Cause-Specific Excess Mortality During the First Two Years of the COVID-19 Pandemic in North-East of Iran: Reiterating the Significance of High-Quality Healthcare Systems

**DOI:** 10.34172/ijhpm.8757

**Published:** 2025-05-21

**Authors:** Seyedeh Vajiheh Kazemian, Maliheh Dadgarmoghaddam, Hamed Tabesh, Amirreza Khajedaluee, Mohammad Khajedaluee

**Affiliations:** ^1^Community Medicine Department, School of Medicine, Mashhad University of Medical Sciences, Mashhad, Iran.; ^2^Department of Medical Informatics, Faculty of Medicine, Mashhad University of Medical Sciences, Mashhad, Iran.; ^3^Sinus and Surgical Endoscopic Research Center, Mashhad University of Medical Sciences, Mashhad, Iran.

**Keywords:** COVID-19 Pandemic, All-Cause Excess Deaths, All-Cause Excess Mortality, Cause-Specific Excess Death, Cause-Specific Excess Mortality, Iran

## Abstract

**Background:**

Excess mortality provides a comprehensive measure to assess the true impact of the disease on mortality rates. This study aimed to quantify excess mortality attributable to COVID-19 in northeastern Iran during the pandemic period (2020–2022).

**Methods:**

This population-based cross-sectional study utilized population and mortality data extracted from electronic systems linked to Mashhad University of Medical Sciences (MUMS). Data analysis was conducted using R Version 4.3.3. A log-linear model was employed to predict expected deaths during the two-year pandemic period, incorporating predictor variables such as the year of interest, the presence of COVID-19, and the population size for each respective year. Excess deaths were calculated as the difference between the expected and observed mortality. Furthermore, by considering the confirmed deaths directly attributed to COVID-19 and the difference between these and the excess deaths, the number of indirect deaths during the pandemic was determined.

**Results:**

The total count of recorded deaths from all causes exceeded the expected deaths by 31.15% (6750 cases) in the first year and 44.74% (10 078 cases) in the second year. The excess deaths were 1.48 and 1.79 times greater than the official reports of COVID-19 for the first and second years, respectively. It was also found that men experienced increased rates of excess deaths in each of the two years. Moreover, urban residents experienced higher rates of excess death in the same years. Based on cause-specific excess mortality, following infectious diseases, cardiovascular diseases (CVDs) accounted for the largest proportion of excess deaths in both years of the pandemic.

**Conclusion:**

The overall mortality burden during the COVID-19 pandemic exceeded the official reports, highlighting the undercounting of the number of direct effects and emphasizing the significance of indirect effects. These findings underscore the importance of preparedness and organization of healthcare systems prior to a pandemic.

## Background

**Key Messages**

**Implications for policy makers**

- Relying solely on official mortality statistics during pandemics may provide an incomplete representation of the true burden of deaths, particularly in the context of limited health system capacity.
- During the pandemic, mortality due to cardiometabolic disorders, unintentional accidents, and suicides exceeded expectations. This finding reveals the fragility of health systems to manage such diseases in pandemics.
- Identifying and focusing on vulnerable populations during pandemics can help reduce the overall mortality burden associated with these events.

**Implications for the public**

Understanding how the COVID-19 pandemic affects public health is very important but given the complexity of the disease’s effects on mortality and its possible underdiagnosis in certain contexts, confirmed COVID-19 deaths alone cannot fully capture the pandemic’s mortality burden. This issue is much more important in low- and middle-income countries that have limited resources. Excess death estimation studies in such a context can be useful for evaluating the effectiveness of health system responses, identifying vulnerable groups, informing the allocation of resources, and providing a platform for better preparation and organization to face possible future pandemics.

The COVID-19 pandemic, deemed the 21st century’s paramount health crisis,^1^ has significantly affected global health and mortality.^2^ Direct impacts include deaths from the disease, often unattributed to COVID-19 due to limited testing capacity, misclassification of death causes,^3^ testing policies, inadequate reporting, healthcare inaccessibility, fragile systems,^4^ or deliberate misinformation.^5^ Indirectly, the pandemic has altered mortality patterns of other diseases through socio-economic shifts, behavioral changes in seeking healthcare,^6^ increased domestic violence, suicides, mental health issues,^7^ and healthcare system burdens causing overcrowding and resource scarcity.^8-10^

 Assessing the COVID-19 pandemic’s public health impact is essential,^11^ but confirmed death counts alone are insufficient to gauge its full effect due to complex mortality influences and underdiagnosis risks,^12^ especially in low-resource settings.^13^ While reported cases are a fraction of actual infections, the Observatório COVID-19 BR, an independent initiative aimed at disseminating quality information on the COVID-19 pandemic in Brazil, suggests 1.21 to 1.41 times more unrecorded deaths per confirmed case.^1^ Literature indicates actual mortality rates maybe 5 to 25 times greater than those reported.^7^

 Excess mortality, not influenced by diagnostic uncertainties or underreporting, is the primary metric for assessing COVID-19’s impact.^14^ Historically used to measure impacts of major events,^15^ it compares observed deaths during the pandemic to expected numbers from historical data, encompassing direct and indirect pandemic effects.^16,17^ It also allows for cross-regional comparisons independent of diagnostic capacities.^18^

 Efforts to quantify COVID-19’s excess mortality, particularly in Iran,^19,20^ show it far exceeds lab-confirmed deaths.^21^ A major study of 74 countries from January 2020 to December 2021 found excess deaths to be triple the 5.94 million reported COVID-19 deaths.^11^

 World Health Organization (WHO) estimates indicate global deaths surpassed expectations by 14.9 million during the pandemic, predominantly in low- and middle-income countries.^22^ This underscores the need to assess the true mortality impact in such nations. Research in Iran has measured excess deaths, but cause-specific excess mortality has not been extensively explored in these studies.^23,24^ Understanding COVID-19’s mortality effects is vital for grasping transmission dynamics, assessing health system responses and resilience,^3,25,26^ and guiding resource allocation decisions.^27^ This study evaluates all-cause and cause-specific mortality in Northeast Iran from March 2020 to March 2022, comparing it to the previous eight years, to determine COVID-19’s true mortality impact.

## Methods

###  Study Design and Study Population 

 This study evaluated excess mortality due to COVID-19 in northeast Iran, examining all-cause and specific-cause deaths (March 20, 2020-March 20, 2022) against the prior 8-year mortality data (March 21, 2012-March 19, 2020), covering Mashhad University of Medical Sciences’ (MUMS’) population within these dates. This population includes 17 out of 34 cities in Razavi Khorasan province located in the northeast of Iran. The names of the covered cities are Mashhad, Torghabeh-Shandiz, Sarkhs, Kalat, Dargaz, Qochan, Chenaran, Fareeman, Kashmar, Bardaskan, Khalilabad, Bakharz, Taybad, Roshtkhwar, Khaf, Golbahar, and Kuohsorkh.

###  Data Source and Data Collection

 The study analyzed two data sets from March 21, 2012, to March 20, 2022. Population data were obtained from the electronic vital statistics registration system, and mortality statistics from the electronic death and classification registration and classification system, both linked to MUMS’ Health Vice-Chancellor. Iran’s health network ensures that electronic registration systems encompass the entire covered area, with information recorded daily by trained staff.

####  Vital Statistics Registration System

 Vital events for the population at rural and urban health centers are daily logged electronically by trained staff. The data is then validated both quantitatively and qualitatively, including cross-referencing with civil registration records by an expert. Following corrections and deduplication, it’s recorded in the vital statistics and classification system and checked for precision. Consequently, the university’s demographic and mortality statistics are updated yearly.^28^

####  Death Registration and Classification System

 The county healthcare center manages death data collection and quality at the county level. An expert reviews data from multiple sources, including hospitals and health centers, for accuracy and eliminates duplicates by cross-referencing with the civil registration. Revised data is then input into the death registration software and integrated into the national system, updating the university’s population death causes annually. Per national guidelines, coding a deceased’s cause of death is a key step before system entry. Coders, possibly medically certified or trained, use the International Classification of Diseases (ICD) and legal standards, to code causes on death certificates.^29^ Currently, death cause classification follows ICD-10.^30^

 Data from March 21, 2012, to March 20, 2022, were sourced from the vital statistics registration system and the death registration and classification system, anonymized and tabulated in Excel. The vital statistics registration system provided annual population numbers by gender and locale (urban/rural), while the death registry system offered yearly death statistics by cause, gender, locale (urban/rural), year of death, and cause of death.

 This study focused on analyzing the fourteen leading causes of mortality within the investigated population, which were identified as the most prevalent during the specified period. These causes are as follows: Diseases of the cardiovascular system (ICD-10: I00-I99), Neoplasms (ICD-10: C00-D48), Diseases of the respiratory system (ICD-10: J00-J99), Unintentional accidents (traffic and non-traffic) (ICD-10: V01-V99, W00, X59, Y40-Y98 (Except Y87.0 and Y87.1)), Endocrine, nutritional and metabolic diseases (ICD-10: E00-E89), Conditions originating in the perinatal period (ICD-10: P00-P96), Diseases of the digestive system (ICD-10: K00-K93), Infectious and parasitic diseases (ICD-10: A00-B99), Diseases of the genitourinary system (ICD-10: N00-N99), Diseases of the nervous system (ICD-10: G00-G99), Congenital malformations, deformations and chromosomal abnormalities (ICD-10: Q00-Q99), Mental, Behavioral and Neurodevelopmental disorders (ICD-10: F01-F99), Violence (ICD-10: X85-Y36 and Y87.1), Suicide (ICD-10: X60-X84 and X87.0), COVID-19 (U07.1, U07.2).

 In the electronic death registration and classification system affiliated with MUMS, the recording and classification of COVID-19 deaths adhere to WHO guidelines.^31^ This study considers lab-confirmed COVID-19 deaths (ICD-10: U07.1) as direct pandemic fatalities.

###  Outcome Measurement 

 Upon extracting and consolidating the primary data, mortality indices were computed both in general and stratified by gender (male/female), residential area (urban/rural), and cause of death. The mortality indicators utilized in this study encompassed the following:

1. Observed death count (Obs): The recorded number of deaths within a specified timeframe.
2. Expected death count (Exp): The anticipated number of deaths during a given timeframe based on historical data.
3. Excess death count: The difference between the observed and expected number of deaths within a specific timeframe.
4. Direct deaths of COVID-19: The officially reported deaths caused by COVID-19 (ICD-10: U07.1) within a designated time period.
5. Indirect deaths of COVID-19: The difference between the excess deaths and the direct deaths specifically caused by COVID-19 within a certain time frame.

Moreover, in addition to the indicators concerning the number of deaths, the rates of deaths per 10 000 individuals were calculated for the aforementioned indices, taking into consideration the population of each year.

###  Statistical Analysis

 Data on population and mortality were analyzed using R 4.3.3. The model aimed to predict annual death counts using the year of interest, COVID-19 status, and corresponding population as predictors. The count data’s suitability for the Poisson distribution led to the use of a log-linear model within the generalized linear model (GLM) framework.

 Given that the number of deaths per population is a count that follows Poisson distribution, a GLM with a log-link function is used, as the response variable in our study is the mortality rate, a log-linear model was utilized as follows: Hence, the general structure of the model was formulated as follows:


logDtPt=β0+β1×Yeart+β2×COVIDt

Where T = Time index for the year of study, t = 1, 2, …, 11.

 D_t _= Number of deaths per population at year i.

 P_t _= Population in the same year

 Year_t_ = Year of study, Year_t_ = 2012, 2013, …., 2022 AP

 COVID_t_ = Status of COVID-19 in the year t COVID_t_ = 0 (for t = 1, 2, …, 9), 1 (for t = 10), 1.26 (for t = 11).

 As, logDtPt=logDt−logPt , “log(*P*_t_)” could be added as an offset in systematic component:

 log(*D*_t_) = *β*_0_ + 1 * log(*P*_t_) + *β*_1 _× *Year*_t_ + *β*_2 _× *COVID*_t_

 In this model, COVID-19 status is a trivalent variable: 0 for pre-pandemic (t = 1, 2, …, 9), 1 for the pandemic’s first year, and 1.26 for the second year. This adjustment in the second year was informed by the ratio of confirmed COVID-19 deaths in the second year relative to those in the first year in the population covered by MUMS.

 To estimate the excess death from each cause across the total population and separately for gender, and regions the predicted values of expected death during the COVID-19 pandemic years were calculated by assuming the absence of COVID-19 and considering COVID_t_ = 0 for pandemic years as follows:

*Excess Death*_t_ = *D*_t_ – Exp(*β*_0_ + 1 * log(*P*_t_) + *β*_1 _× *Year*_t_)

 In order to separate the excess death of all causes into direct and indirect deaths of COVID-19, by differentiating the count of definite COVID-19 deaths as direct deaths, the count of indirect deaths attributed to COVID-19 related to the years coinciding with the pandemic was obtained. Moreover, we computed the death rate per 10 000 individuals using the mortality data (expected and observed). Subsequently, the mortality metrics were displayed in tabular and graphical formats.

 During data analysis, the GLM model’s suitability was assessed by deviance and Akaike’s information criterion (AIC) values. A model is considered better if it has a significant decrease in residual deviance relative to null deviance and a lower AIC value.

## Results

 Based on data extracted from the MUMS Health Vice-Chancellor’s vital statistics and classification registration system, the population under the purview of this university in March 2022 totaled 5 132 208 individuals, comprising 2 583 823 (50.35%) males and 2 548 385 (49.65%) females residing across 17 cities. Within this demographic, 4 050 401 individuals (78.92%) resided in urban regions, while 1 081 807 individuals (21.08%) inhabited rural areas.

###  All-Cause Excess Mortality

 During the two-year period encompassing the COVID-19 pandemic within the analyzed population, there was a notable increase in observed deaths compared to expected deaths. Specifically, a 31.15% rise with 6750 (95% CI: 6519-7221) cases were reported the first year, while the second year recorded a 44.74% surge with 10 078 (95% CI: 9919-10 679) cases. This discrepancy between observed and expected deaths, representing the excess mortality from all causes during the COVID-19 pandemic, was calculated at 13.27 per 10 000 individuals in the initial year and 19.64 per 10 000 in the subsequent year of the pandemic. The graphical representation in Figure illustrates the fluctuation in all-cause mortality (both observed and expected) throughout the examined years.

**Figure:**
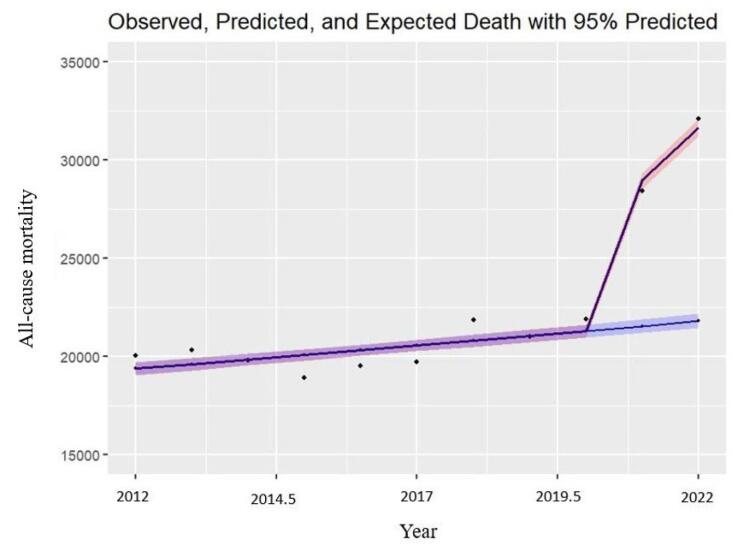
The Trend of Annual All-Cause Mortalities (Observed and Expected With 95% CI) During the Years Under Study (March 2012 to March 2022). Abbreviation: CI, confidence interval.

 During the two-year period examined, COVID-19 was directly responsible for 67.35% (4546 cases) and 55.71% (5615 cases) of the excess deaths from all causes, with the remaining deaths demonstrating an indirect association with the pandemic. Consequently, the estimated number of indirect deaths linked to COVID-19 during these two years stood at 2204 cases (4.33 per 10 000) and 4463 cases (8.70 per 10 000), respectively. Notably, the excess mortality in the initial and subsequent years of the pandemic exceeded official reports (based on confirmed COVID-19 cases) by 1.48 and 1.79 times. Table 1 outlines the numbers and rates of deaths from all causes (observed, expected, excess), as well as direct and indirect deaths attributed to COVID-19 for the concurrent two-year period with the pandemic.

**Table 1:** All-Cause Mortalities and Direct and Indirect Mortalities of COVID-19 During the Years Under Study (March 2020 to March 2022)

**Years **	**Type of Deaths **	**Number of Deaths(Rate Per 10 000)**				
		**Male**	**Female**	**Urban**	**Rural**	**Total**
First year	Observed	16 488 (64.35)	11 934 (47.30)	22 528 (56.01)	5894 (55.42)	28 422 (55.89)
	Expected	11 287 (44.05)	9044 (35.85)	16 090 (40.01)	4544 (42.73)	21 672 (42.62)
	Excess	5201 (20.30)	2890 (11.45)	6438 (16.01)	1350 (12.70)	6750 (13.27)
	Direct COVID-19	2719 (10.61)	1827 (7.24)	3798 (9.44)	748 (7.03)	4546 (8.94)
	Indirect COVID-19	2482 (9.69)	1063 (4.21)	2640 (6.56)	602 (5.66)	2204 (4.33)
Second year	Observed	18 038 (69.81)	14 072 (55.22)	26 451 (65.31)	5659 (52.31)	32 110 (62.57)
	Expected	11 436 (44.26)	9222 (36.19)	16 426 (40.55)	4656 (43.04)	22 032 (42.93)
	Excess	6602 (25.55)	4850 (19.03)	10 025 (24.75)	1003 (9.27)	10 078 (19.64)
	Direct COVID-19	3086 (11.94)	2529 (9.92)	4914 (12.13)	701 (6.48)	5615 (10.94)
	Indirect COVID-19	3516 (13.61)	2321 (9.11)	5111 (12.62)	302 (2.79)	4463 (8.70)
Residual deviance		118.63	143.34	116.23	58.28	241.34
AIC		252.61	274.11	253.45	182.2	381.47

Abbreviation: AIC, Akaike’s information criterion.

 Based on gender, the excess mortality due to all causes among males during the initial and subsequent years of the pandemic stood at 5201 cases (20.30 per 10 000) and 6602 cases (25.55 per 10 000), respectively, while for females, the numbers were 2890 cases (11.45 per 10 000) and 4850 cases (19.03 per 10 000), respectively. These findings indicate that the excess death rate among males was 1.77 times higher in the first year and 1.34 times higher in the second year compared to females. Consequently, men exhibited a greater excess mortality rate than women during the COVID-19 pandemic years.

 According to the geographical location of residence, the excess mortality rate for all causes in urban regions during the initial and subsequent years of the pandemic amounted to 6438 cases (16.01 per 10 000) and 10 025 cases (24.75 per 10 000), respectively, whereas rural areas reported 1350 cases (12.70 per 10 000) and 1003 cases (9.27 per 10 000) for the same periods. Notably, urban areas exhibited a higher excess mortality rate in both years coinciding with the COVID-19 pandemic. Consequently, the excess death rate in urban regions was 1.26 times higher in the first year and 2.67 times higher in the second year compared to rural areas.

###  Cause-Specific Excess Mortality

 In the studied population, mortality rates for specific causes—such as infectious diseases (including COVID-19), cardiovascular diseases (CVDs), endocrine, nutritional, and metabolic disorders (ENMDs), unintentional accidents, and suicides—exceeded expected levels during the COVID-19 pandemic years. Conversely, observed mortality for causes like violence-related deaths, mental disorders, neurological conditions, genitourinary diseases, and perinatal issues was lower than expected. Notably, the comparison between observed and expected deaths for neoplasms and respiratory diseases (excluding COVID-19) in the initial and subsequent years of the pandemic revealed distinct trends. Specifically, neoplasm-related deaths exceeded expectations in the first year but decreased significantly in the second year, whereas respiratory disease deaths were below expectations in the first year but notably increased in the second year. Table 2 provides detailed data on cause-specific mortality rates (observed, expected, and excess) over the two years coinciding with the COVID-19 pandemic.

**Table 2:** Number of Cause-Specific Mortalities During the Years Coinciding With the Pandemic (March 2020 to March 2022)

**Cause of Deaths**	**First Year of the Pandemic** **Number (Rate Per 10 000)**			**Second Year of the Pandemic ** **Number (Rate Per 10 000)**			**Residual Deviance**	**AIC**
	**Observed**	**Expected**	**Excess**	**Observed**	**Expected**	**Excess**		
Cardiovascular system	9000	7559	1441	9709	7722	1987	193.69	322.61
	(17.70)	(14.86)	(2.84)	(18.92)	(15.05)	(3.87)		
Neoplasms	3675	3580	95	3512	3,768	-256	36.47	155.33
	(7.23)	(7.04)	(0.19)	(6.84)	(7.34)	(-0.50)		
Respiratory system (Except COVID-19)	2019	2080	-61	2416	2146	270	328.33	442.52
	(3.97)	(4.09)	(-0.12)	(4.71)	(4.18)	(0.53)		
Unintentional accidents (traffic and non-traffic)	1517	1,487	30	1641	1418	223	80.49	192.85
	(2.98)	(2.92)	(0.06)	(3.2)	(2.76)	(0.44)		
Endocrine, nutritional, and metabolic diseases	1773	1294	479	1608	1399	209	137.73	243.61
	(3.49)	(2.54)	(0.95)	(3.13)	(2.73)	(0.40)		
Conditions originating in the perinatal period	591	731	-140	597	704	-107	109.84	213.07
	(1.16)	(1.44)	(-0.28)	(1.16)	(1.37)	(-0.21)		
Digestive system	609	661	-52	701	696	5	38.17	138.35
	(1.20)	(1.30)	(-0.10)	(1.37)	(1.36)	(0.01)		
Infectious and parasitic diseases (including COVID-19)	7079	647	6432	9557	692	8865	49.13	153.71
	(13.92)	(1.27)	(12.65)	(18.62)	(1.35)	(17.27)		
Genitourinary system	467	557	-90	494	584	-90	69.46	168.52
	(0.92)	(1.10)	(-0.18)	(0.96)	(1.14)	(-0.18)		
Nervous system	417	456	-39	423	482	-59	24.37	120.17
	(0.82)	(0.90)	(-0.08)	(0.82)	(0.94)	(-0.12)		
Congenital malformations, deformations and chromosomal abnormalities	250	393	-143	213	382	-169	27.68	123.54
	(0.49)	(0.77)	(-0.28)	(0.42)	(0.74)	(-0.32)		
Mental, behavioral, and neurodevelopmental disorders	203	220	-17	58	242	-184	53.99	139.00
	(0.40)	(0.43)	(-0.03)	(0.11)	(0.47)	(-0.36)		
Violence	137	161	-24	123	151	-28	35.86	123.53
	(0.27)	(0.32)	(-0.05)	(0.24)	(0.29)	(-0.05)		
Suicide	226	185	41	230	186	44	29.54	117.43
	(0.44)	(0.36)	(0.08)	(0.45)	(0.36)	(0.09)		

Abbreviation: AIC, Akaike’s information criterion.

 Throughout both pandemic years, after deaths from infectious diseases (including COVID-19), deaths from CVDs accounted for the highest number of excess deaths. Deaths attributed to CVDs, encompassing various subcategories under ICD-10 such as acute rheumatic fever, chronic rheumatic heart diseases, hypertensive diseases, ischemic heart diseases, pulmonary heart disease, and diseases of pulmonary circulation, cerebrovascular diseases, diseases of arteries, arterioles and capillaries, diseases of veins, lymphatic vessels and lymph nodes, surpassed expected levels by 19.06% (1441 cases) in the first year and 25.73% (1987 cases) in the second year of the pandemic.

 Following CVDs, ENMDs emerged as the next most prevalent cause of mortality in the initial year of the pandemic. Deaths attributed to ENMDs, encompassing specific ICD-10 subcategories such as disorders of the thyroid gland, diabetes mellitus, other disorders of glucose regulation and pancreatic internal secretion, disorders of other endocrine glands, malnutrition, other nutritional deficiencies, overweight, obesity, and other hyperalimentation, metabolic disorders, exhibited a respective excess of 37.01% (479 cases) and 14.94% (209 cases) over expected deaths in the two pandemic years.

 In the subsequent year of the pandemic, respiratory diseases followed CVDs in terms of mortality. Deaths attributed to respiratory diseases, encompassing various ICD-10 subgroups including acute upper respiratory infections, influenza and pneumonia, other acute lower respiratory infections, other diseases of the upper respiratory tract, chronic lower respiratory diseases, lung diseases due to external agents, other respiratory diseases mainly affecting the interstitium, suppurative and necrotic conditions of the lower respiratory tract, other diseases of the pleura, were 2.93% (61 cases) below expected levels in the first pandemic year but increased by 12.58% (270 cases) beyond expected levels in the subsequent pandemic year.

 The greatest increase in observed mortality compared to expected mortality in both years of the pandemic was related to infectious diseases, followed by ENMDs (37.02%), suicide (22.16%), and CVDs (19.06%) in the first year of the pandemic, respectively, and in the second year of the epidemic, it occurred for CVDs (25.73%), suicide (23.66%) and unintentional accidents (15.73%), respectively.

## Discussion

 The present study aimed to analyze mortality during the pandemic period (20 March 2020 to 20 March 2022) in comparison to past trends (21 March 2012 to 19 March 2020) and estimate excess all-cause mortality and cause-specific excess deaths in Northeast Iran. This would enable the real impact of this disease on mortality in the region to be determined.

 The findings showed a 31.15% and 44.74% increase in mortality during the initial two-year period of the pandemic, aligning with other studies. Variations in these numbers are attributed to the quality of mortality data registration and estimation methods used for expected deaths in a non-pandemic scenario. A related study using seven-year historical data (2015-2019) found a 35.15% and 51.33% rise in mortality for 2020-2021 and 2021-2022, respectively.^23^ Nucci et al observed a 1.14 to 1.40-fold increase in Brazil’s mortality rate during 2020-2021,^32^ while the 2020 study in Florida and Ohio estimated a 1.17- and 1.15-times higher mortality, respectively.^33^ These discrepancies, termed excess deaths, likely stem from various causes, including COVID-19’s direct and indirect impacts. To reduce future excess deaths during pandemics, it is crucial to prioritize strengthening healthcare infrastructure in urban areas and enhancing emergency care capacity in rural areas.

 The study found pandemic excess deaths to be 1.48 and 1.79 times the official COVID-19 deaths in the first and second years, respectively. Such a result has also been reported in other studies. For instance, a global study tracking excess mortality during the COVID-19 pandemic indicated that Iran’s excess deaths in 2020 were 2.42 times greater than the officially reported COVID-19 deaths.^27^ Similarly, another study in Iran found that excess deaths over the first two years of the pandemic were 1.76 times higher than confirmed COVID-19 deaths.^19^ In Sanmarchi and colleagues’ study of 67 countries, excess deaths in most countries were found to be higher than confirmed deaths from COVID-19.^34^ Discrepancies between excess deaths and official reports of COVID-19 may stem from COVID-19 death underreporting due to inadequate lab facilities, unrecognized clinical signs, and socio-political factors.^35^ Furthermore, these discrepancies can be attributed to the indirect effects of the pandemic on mortality. The indirect effects of the pandemic can be attributed to the excessive pressure placed on healthcare systems, which in turn leads to poorer outcomes for other acute diseases and impaired diagnosis and management of chronic diseases.^36^ Additionally, broader socio-economic impacts of the pandemic, such as unemployment, social isolation, and loneliness, are other indirect effects of the pandemic.^37^

 Analysis reveals that during the pandemic’s first and second years, 67.35% and 55.71% of excess deaths were directly due to COVID-19, respectively, with the rest being indirect consequences. Study outcomes differ by time and context, influenced by health systems’ testing and crisis management capacities. For example, early in Iran’s outbreak, 49.10% of excess deaths were COVID-related,^20^ compared to 51.00% in Portugal.^38^

 During the COVID-19 pandemic, men had higher excess death rates than women. Studies in Iran,^19^ and Brazil,^8^ confirm this trend, possibly due to men’s greater occupational exposure and non-compliance with health measures. This necessitates targeted infectious disease interventions, especially immunization. The observed mortality disparity among genders warrants further investigation beyond COVID-19 contexts.

 This study found urban areas had higher excess death rates than rural areas during the two-year pandemic, despite better healthcare access. Contrarily, a study in Iran reported higher rural excess deaths,^24^ while one in Minnesota found higher non-rural excess deaths,^39^ aligning with our findings. Furthermore, the existence of differences in the estimate of excess death rate based on the area of residence has also been reported in other studies, including the study of Ackley et al, in which excess mortality was reported in some provinces in urban areas and in some provinces in non-urban areas.^37^ Urban overcrowding likely exacerbated the pandemic spread, increasing mortality. Urban lifestyle, pollution, and non-communicable diseases may also amplify excess deaths associated with the pandemic, as seen in rising urban death rates.

 During the pandemic, mortality from causes like CVDs, ENMDs, unintentional accidents, and suicides exceeded expectations. In contrast, deaths from violence, mental, neurological, genitourinary diseases, and perinatal conditions were below anticipated levels. Notably, neoplasms and respiratory diseases (excluding COVID-19) showed varying patterns across the two years. Studies, including Pirayesh and colleagues in Iran, noted increased mortality for endocrine and CVDs, while genitourinary and certain perinatal conditions showed declines.^24^ Norwegian research (2020-2022) indicated higher-than-expected deaths from CVDs and malignant tumors, but fewer from non-COVID respiratory diseases and dementia.^40^ In England and Wales, more deaths from dementia, Alzheimer’s, diabetes, Parkinson’s, and heart diseases were reported in the first pandemic year, but fewer from pneumonia, influenza, stroke, and accidents.^11^ An English study found CVDs mortality 33.00% higher, acute respiratory infections 43.00% higher, diabetes 35.00% higher, and liver diseases 19.00% higher than expected.^36^ These findings suggested the pandemic’s impact on healthcare systems and containment measures like quarantine could influence mortality from various causes, highlighting the importance of recognizing such effects for resource allocation and planning. The rise in mortality from certain causes during the pandemic has revealed the preexisting fragility of health systems to manage such diseases.^41,42^

 According to the findings of the present study, during the pandemic, in addition to infectious, CVDs, and ENMDs, suicide also experienced the highest increase in observed mortality compared to the expected deaths in both investigated years. This finding aligns with a global meta-analysis that indicated heightened rates of suicidal ideation, attempted suicide, and self-harm during the COVID-19 pandemic compared to pre-pandemic studies.^43^ Similar results were reported in other studies, including those by Bridge et al^44^ and Lee et al.^45^ This increase can be attributed to the profound psychological and social effects of the COVID-19 pandemic, such as diminished social cohesion and interaction during the pandemic, along with the fears it generated.^46^ Based on this, early and preventive social care, as well as the implementation of effective suicide prevention strategies during the pandemic, are recommended,^47^ including the provision of evidence-based remote interventions (eg, telephone or digital) that can enhance population mental health.^48^

 One of the key strengths of this study is the utilization of modeling techniques to estimate expected death, as well as to determine the extent of excess mortality due to specific causes. It leverages an 80-year data set and data from an electronic vital statistics registration system and electronic death registration and classification system, significantly enhancing data precision and accuracy. Furthermore, the study’s strength lies in the fact that all those issuing death certificates underwent a workshop on recording the causes of death at the outset of the COVID-19 pandemic. Furthermore, based on the study design, the generalizability of the findings for Razavi Khorasan province can be inferred. This province, with a population of over 5 million people, is situated in the north-east of Iran and has the second-largest population in the country.

 One potential limitation of the current study is that before the prevalence of the COVID-19 pandemic, the medical professionals responsible for issuing death certificates lacked the requisite expertise to diagnose the underlying cause of death (COVID-19 or an existing condition). However, the registration workshop promptly addressed this issue by accurately recording the causes of death immediately after the release of WHO guidelines, thereby ensuring the reliability of the data. One additional limitation of this study is the absence of information regarding variables that may have evolved over time, including access to healthcare services and shifts in the socioeconomic status of the population under investigation, this resulted in constraints on further analysis. Variations in these factors could have impacted the mortality trends, either favorably or unfavorably, and this influence may be especially pronounced when examining cause-specific mortality. Furthermore, a notable limitation within the death registration and classification system is the potential for misclassification of causes of death. Nevertheless, this study aimed to select a period during which the electronic system for registering and classifying causes of death was fully operational, and the personnel had received adequate training in this area to mitigate this issue to the greatest extent possible. Also, one limitation of the study was the inability to conduct a sensitivity analysis because of insufficient information on unrecorded COVID cases in electronic systems.

## Conclusion

 During the COVID-19 pandemic’s two-year span, excess mortality was 1.48 and 1.79 times the official COVID-19 reports in the first and second years, respectively, indicating underreported pandemic mortality, and hospital strain. The findings underscored the system’s lack of readiness for precise diagnosis and prompt action, stressing the critical necessity to allocate resources towards research, enhancing healthcare infrastructure, and training personnel to effectively address emerging diseases. The pandemic’s indirect effects, including healthcare deficits and psychological and social impacts, exacerbated mortality from other causes. These findings underscore the importance of healthcare system readiness to manage pandemics’ direct and indirect effects while maintaining other healthcare services. Prioritizing vulnerable populations, notably those affected by CVDs, ENMDs, and respiratory diseases, could mitigate excess deaths associated with the pandemic. Recognizing the pandemic’s mortality impact is vital for addressing immediate excess death factors and preparing for future pandemics by monitoring long-term complications and mortality. This information acts as a crucial resource for monitoring the forthcoming stages of the ongoing pandemic and facilitates the evaluation of both local and national mitigation strategies. Such insights can contribute to the formulation of effective health policies moving forward.

## Acknowledgement

 We would like to express our gratitude to the Vice-Chancellor of Research of MUMS and the Vice-Chancellor of Health of MUMS for their cooperation in this study.

## Ethical issues

 This study was approved by the Biomedical Research Ethics Committee part of MUMS (Ethical approval code: IR.MUMS.REC.1401.051), and research was performed following relevant regulations with anonymized data. The research was conducted in compliance with applicable ethical guidelines and regulations, utilizing anonymized data to ensure confidentiality and adherence to ethical standards.

## Conflicts of interest

 Authors declare that they have no conflicts of interest.

## Data availability statement

 The data that support the findings of this study are available from MUMS but restrictions apply to the availability of these data, which were used under license for the current study, and so are not publicly available. Data are however available from the authors upon reasonable request and with permission of MUMS.

## References

[1] Saraceni V, Cruz OG, Cavalcante JR (2023). Excess mortality from all causes during the COVID-19 pandemic in the city of Rio de Janeiro, Brazil. Rev Bras Epidemiol.

[2] Thong PM, Chong HT, Chang AJW, Ong CWM (2023). COVID-19, the escalation of diabetes mellitus and the repercussions on tuberculosis. Int J Infect Dis.

[3] Acosta E. Global Estimates of Excess Deaths from COVID-19. London, UK: Nature Publishing Group; 2023. 10.1038/d41586-022-04138-w 36517677

[4] Aborode AT, Hasan MM, Jain S (2021). Impact of poor disease surveillance system on COVID-19 response in Africa: time to rethink and rebuilt. Clin Epidemiol Glob Health.

[5] Fantin R, Barboza-Solís C, Hildesheim A, Herrero R (2023). Excess mortality from COVID 19 in Costa Rica: a registry-based study using Poisson regression. Lancet Reg Health Am.

[6] Han L, Zhao S, Li S (2023). Excess cardiovascular mortality across multiple COVID-19 waves in the United States from March 2020 to March 2022. Nat Cardiovasc Res.

[7] Gmanyami JM, Jarynowski A, Belik V, Lambert O, Amuasi J, Quentin W (2024). Excess mortality during the COVID-19 pandemic in low-income and lower middle-income countries: protocol for a systematic review and meta-analysis. BMJ Open.

[8] Colonia SR, Cardeal LM, de Oliveira RA, Trinca LA (2023). Assessing COVID-19 pandemic excess deaths in Brazil: years 2020 and 2021. PLoS One.

[9] Kontopantelis E, Mamas MA, Deanfield J, Asaria M, Doran T (2021). Excess mortality in England and Wales during the first wave of the COVID-19 pandemic. J Epidemiol Community Health.

[10] Rhee C (2022). Deconstructing improvements and hospital variation in COVID-19 mortality rates during the early pandemic wave: the effects of wave evolution and advances in testing, treatment, and hospital care quality. BMJ Qual Saf.

[11] Laliotis I, Stavropoulou C, Ceely G, Brett G, Rushton R (2023). Excess deaths by cause and place of death in England and Wales during the first year of COVID-19. Health Econ.

[12] Matz M, Allemani C, van Tongeren M (2022). Excess mortality among essential workers in England and Wales during the COVID-19 pandemic. J Epidemiol Community Health.

[13] Murhekar MV, Bhatnagar T, Thangaraj JW (2021). SARS-CoV-2 seroprevalence among the general population and healthcare workers in India, December 2020-January 2021. Int J Infect Dis.

[14] Ruhm CJ (2022). Excess deaths in the United States during the first year of COVID-19. Prev Med.

[15] Weinberger DM, Chen J, Cohen T (2020). Estimation of excess deaths associated with the COVID-19 pandemic in the United States, March to May 2020. JAMA Intern Med.

[16] Alicandro G, Gerli AG, Centanni S, Remuzzi G, La Vecchia C (2023). Excess total mortality in Italy: an update to February 2023 with focus on working ages. Med Lav.

[17] Jain V, Clarke J, Beaney T (2022). Association between democratic governance and excess mortality during the COVID-19 pandemic: an observational study. J Epidemiol Community Health.

[18] Ledesma JR, Isaac CR, Dowell SF (2023). Evaluation of the Global Health Security Index as a predictor of COVID-19 excess mortality standardised for under-reporting and age structure. BMJ Glob Health.

[19] Ebrahimoghli R, Abbasi-Ghahramanloo A, Moradi-Asl E, Adham D (2023). The COVID-19 pandemic’s true death toll in Iran after two years: an interrupted time series analysis of weekly all-cause mortality data. BMC Public Health.

[20] Safavi-Naini SAA, Farsi Y, Alali WQ, Solhpour A, Pourhoseingholi MA (2022). Excess all-cause mortality and COVID-19 reported fatality in Iran (April 2013-September 2021): age and sex disaggregated time series analysis. BMC Res Notes.

[21] Riou J, Hauser A, Fesser A, Althaus CL, Egger M, Konstantinoudis G (2023). Direct and indirect effects of the COVID-19 pandemic on mortality in Switzerland. Nat Commun.

[22] Lewnard JA, B CM, Kang G, Laxminarayan R (2023). Attributed causes of excess mortality during the COVID-19 pandemic in a south Indian city. Nat Commun.

[23] Esmaeilzadeh N, Hoseini SJ, Jafari Nejad Bajestani M (2023). Excess mortality in Northeast Iran caused by COVID-19: neglect of offset community transformations of health. Asian Pac J Trop Med.

[24] Pirayesh Z, Riahi SM, Bidokhti A, Kazemi T (2023). Evaluation of the effect of the COVID-19 pandemic on the all-cause, cause-specific mortality, YLL, and life expectancy in the first 2 years in an Iranian population-an ecological study. Front Public Health.

[25] Paul E, Brown GW, Kalk A, Van Damme W, Ridde V, Sturmberg J (2022). “When my information changes, I alter my conclusions. ” What can we learn from the failures to adaptively respond to the SARS-CoV-2 pandemic and the under preparedness of health systems to manage COVID-19? Int J Health Policy Manag.

[26] Bozorgmehr K, Zick A, Hecker T (2022). Resilience of health systems: understanding uncertainty uses, intersecting crises and cross-level interactions: Comment on “Government actions and their relation to resilience in healthcare during the COVID-19 pandemic in New South Wales, Australia and Ontario, Canada”. Int J Health Policy Manag.

[27] Karlinsky A, Kobak D (2021). Tracking excess mortality across countries during the COVID-19 pandemic with the World Mortality Dataset. Elife.

[28] Vice President of Health M, Network Management Center, Network System Information and Statistics Department. Instructions for completing the vital poster of rural and urban areas [Persian]. https://www.medsab.ac.ir/uploads/Final-Dastoramalzij_31_04_94_51706.pdf. Accessed January 5, 2024.

[29] Vice President of Health M, Network Management Center, Network System Information and Statistics Department. Guide to the death registration and classification system [Persian]. https://tinylink.info/Y8Cr. Accessed January 5, 2024.

[30] 2024 ICD-10-CM Codes. https://www.icd10data.com/ICD10CM/Codes. Accessed January 5, 2024.

[31] World Health Organization (WHO). International Guidelines for Certification and Classification (CODING) of COVID-19 as Cause of Death. https://www.who.int/docs/default-source/classification/icd/covid-19/guidelines-cause-of-death-covid-19-20200420-en.pdf. Accessed January 5, 2024.

[32] Nucci LB, Enes CC, Ferraz FR, da Silva IV, Rinaldi AE, Conde WL (2023). Excess mortality associated with COVID-19 in Brazil: 2020-2021. J Public Health (Oxf).

[33] Quast T, Andel R (2021). Excess mortality associated with COVID-19 by demographic group: evidence from Florida and Ohio. Public Health Rep.

[34] Sanmarchi F, Golinelli D, Lenzi J (2021). Exploring the gap between excess mortality and COVID-19 deaths in 67 countries. JAMA Netw Open.

[35] Ferrara P, Dallagiacoma G, Alberti F, Gentile L, Bertuccio P, Odone A (2022). Prevention, diagnosis and treatment of cervical cancer: a systematic review of the impact of COVID-19 on patient care. Prev Med.

[36] Pearson-Stuttard J, Caul S, McDonald S, Whamond E, Newton JN (2024). Excess mortality in England post COVID-19 pandemic: implications for secondary prevention. Lancet Reg Health Eur.

[37] Ackley CA, Lundberg DJ, Ma L, Elo IT, Preston SH, Stokes AC (2022). County-level estimates of excess mortality associated with COVID-19 in the United States. SSM Popul Health.

[38] Vieira A, Peixoto VR, Aguiar P, Abrantes A (2020). Rapid estimation of excess mortality during the COVID-19 pandemic in Portugal-beyond reported deaths. J Epidemiol Glob Health.

[39] McCoy RG, Campbell RL, Mullan AF (2022). Changes in all-cause and cause-specific mortality during the first year of the COVID-19 pandemic in Minnesota: population-based study. BMC Public Health.

[40] Raknes G, Fagerås SJ, Sveen KA, Júlíusson PB, Strøm MS (2024). Excess non-COVID-19 mortality in Norway 2020-2022. BMC Public Health.

[41] Vamos EP, Khunti K (2022). Indirect effects of the COVID-19 pandemic on people with type 2 diabetes: time to urgently move into a recovery phase. BMJ Qual Saf.

[42] Rachamin Y, Meyer MR, Rosemann T, Grischott T (2023). Impact of the COVID-19 pandemic on elective and emergency inpatient procedure volumes in Switzerland - a retrospective study based on insurance claims data. Int J Health Policy Manag.

[43] Dubé JP, Smith MM, Sherry SB, Hewitt PL, Stewart SH (2021). Suicide behaviors during the COVID-19 pandemic: a meta-analysis of 54 studies. Psychiatry Res.

[44] Bridge JA, Ruch DA, Sheftall AH (2023). Youth suicide during the first year of the COVID-19 pandemic. Pediatrics.

[45] Lee WE, Woo Park S, Weinberger DM (2023). Direct and indirect mortality impacts of the COVID-19 pandemic in the United States, March 1, 2020 to January 1, 2022. Elife.

[46] Sher L (2020). The impact of the COVID-19 pandemic on suicide rates. QJM.

[47] Martínez-Alés G, Szmulewicz A, López-Cuadrado T, Morrison CN, Keyes KM, Susser ES (2023). Suicide following the COVID-19 pandemic outbreak: variation across place, over time, and across sociodemographic groups A systematic integrative review. Curr Psychiatry Rep.

[48] Gunnell D, Appleby L, Arensman E (2020). Suicide risk and prevention during the COVID-19 pandemic. Lancet Psychiatry.

